# The Army Canteen

**Published:** 1905-12

**Authors:** 


### The Army Canteen.

There can be no doubt but that the great majority of the medical profession are strongly in favor of any and all means that will restrict or abolish alcoholism. More than any other class of men are they familiar with the diseases, suffering and abject misery and want produced by this one cause. On the other hand, their studies have enabled them to know that, however much to be desired, a total discontinuance of the stimulant is not practicable, and it is with the practical aspect of this problem that the practicing physician has to deal. All attempts to force abstemiousness upon a community have met with failure, and a failure often of a most humiliating character. The devotees of Bacchus has simply transferred his shrine to another community, visiting at more or less frequent intervals, becomes his own dispenser, or, worst of all, resorts to so-called medicinal bitters and tonics, the basis of which is usually an alcohol of the most destructive character.

The abolition of the army canteen, which was the result of a congressional campaign on the part of the good, zealous but misguided women of the W. T. C. U., has unquestionably not had the desired effect of diminishing intemperance among the soldiers. Quite the contrary. Reports from the Surgeon General's office show that previous to the establishment of the canteen, admissions to sick report numbered about 64 per thousand. With the canteen this large proportion was lowered, at first rapidly and then more slowly, to 19 per thousand, or slightly less than 2 per cent of all cases—extremely small when we consider the manner of living of the soldier in time of peace, the ease with which he may obtain a pass outside the post limits, and the temptations that immediately waylay him. With the passage of the anti-canteen law, saloons situated just outside the post reservations have sprung up like mushrooms, and the result has been a prompt increase in the number of cases of alcoholism. The latest report shows a total of almost 3 per cent, and this proportion is gradually increasing. Among post and department commanders there has been in consequence an almost unanimous recommendation for the repeal of the anti-canteen law as the only method by which the increasing drunkenness can be limited. Whether their recommendation will have any effect is a question. It seems to be a habit of this government to employ experts at good salaries for every department, and then the mass of Congressmen, absolutely ignorant of the measures upon which they are voting, and swayed only by the question of expediency, i. e., votes, pass a ruling diametrically opposed to the advice of their paid experts. It has been often stated in the past few months that many of the temperance advocates instrumental in the passage of the anti-canteen bill have become convinced that its enforcement has not been a practical success, and that they will do nothing to prevent its repeal. With that pressure removed Congress will probably again be able to combine wisdom with "expediency" and restore to the soldier his former privileges.—*Lancet-Clinic.*

---

In an editorial on "The Public Schools and the Public Health," in the *Medical Examiner and Practitioner,* reference is made to a recent address by Dr. J. S. Lankford, of the Board of Education of San Antonio, Texas, in which the doctor contends that if a serious intention exists to utilize the hygienic information acquired during the last few years, it can be done only by co-operation with school children and their teachers. His opinion seems to be that by beginning sufficiently early in life certain thought habits can be enforced upon the children which they will retain and transmit, so that the next generation will do, as by instinct, those things which today the present generation does only by compulsion. In San Antonio the children of the public schools, guided by their teachers, have done what the adults would not even raise their hands to do—the children practically exterminated the *stegomyia.* Object lessons were given to the children, the mosquito was permitted to develop through all its stages, of course in confinement, while the stereopticon and limelight showed the development and life history of the parasite. Dr. Lankford believes that San Antonio will escape an epidemic of the yellow fever, and should it do so the credit, he insists, will belong to the children and not to their parents.—*Literary Digest.*

---

THE Medical Society of the County of New York did well when it recently passed resolutions denouncing those newspapers that publish quack advertisements fitly described as "filthy." It is not only the "filth" of such advertisements, but also their preposterous lies, against which the indignation of all decent persons should be directed, and it does seem as if some means might be found to bring the offending newspapers to a sense of the enormity of aiding and abetting such birds of prey as the quacks.—*New York Medical Journal.*

